# Special Issue “Molecular and Cellular Mechanisms of Action of Markers of Tissue Degeneration”

**DOI:** 10.3390/ijms23126358

**Published:** 2022-06-07

**Authors:** Carlos Gutierrez-Merino, Ana M. Mata

**Affiliations:** 1 Instituto de Biomarcadores de Patologías Moleculares, Universidad de Extremadura, 06006 Badajoz, Spain; anam@unex.es; 2 Departamento de Bioquímica y Biología Molecular, Facultad de Ciencias, Universidad de Extremadura, 06006 Badajoz, Spain

Tissue degeneration is an event shared by many, if not all, age-related pathologies. Brain degeneration is associated with neurological disorders, whose incidence largely increases with human aging. Neurodegenerative diseases associated with aging are increasingly affecting more human beings as their lifespan is expanded, and there is a need for highly efficient therapies against them. These diseases develop from early stages, with mild brain and neurological damage, to advanced stages with severe and widespread brain damage, and also with a large loss of neurological functions that are essential to self-care and the maintenance of a non-dependent human life. Therefore, the identification of biomarkers of initial events and the subsequent propagation of brain and neurological damage should allow for the improvement of early diagnoses of these neurodegenerative disorders. In addition, a better knowledge of the initial molecular mechanisms that foster the development of neurodegenerative diseases is needed to develop more efficient therapeutic treatments and attenuate their incidence in the human population or, at least, to block their advancement into the later stages associated with severe neurological dysfunctions.

In this Special Issue, Pérez et al. [1] performed a brief account of protein biomarkers for the diagnosis of Alzheimer’s disease (AD), one of the most prevalent aging-related neurodegenerative disorders, at different stages of neurodegeneration. The authors comment on risk factors to accelerate aging, and on the molecular and cellular markers that appear at different stages of the disease, before or once the clinical symptoms become evident. These markers are present in biological fluids or could be identified by noninvasive imaging techniques, such as positron emission tomography (PET) using different radiopharmaceutical tracers. This review is largely focused on Tau phosphorylation and specific phosphorylation signatures as early biomarkers of AD. The authors remark a soluble phosphorylated Tau signature (P-Tau 217 and P-Tau 181), which could be induced by amyloid β (Aβ)-activation of the protein kinase cdk5 during very early (asymptomatic) stages of the disease. These phosphorylated forms of Tau are correlated with the development of amyloid plaques at earlier stages of the disease, before neuronal atrophy and brain metabolism changes are detected. They also noted that neuronal atrophy is accompanied by P-Tau 205/P-Tau 202; later on, Tau tangles develop and total Tau increases in the brains of AD patients. Finally, the authors briefly mention other putative AD markers found in cerebrospinal fluid, such as neurofilament light-chain NF-L and CX3CL1 (fractalkine), and the presence of nucleic acids in the cerebrospinal fluid or blood.

The work of Mroczko et al. [2] focuses on protein misfolding in amyloidosis and tauopathies characterized by fibrillar Aβ deposition and neurofibrillary tangles. The authors first highlighted the pathogenic roles of Aβ oligomers (AβOs) and soluble Tau oligomers (TauOs) produced in the early stages of these neurodegenerative diseases, including AD. Then, they analyze in the molecular mechanisms that lead to Tau misfolding and aggregation in more depth, as well as hyperphosphorylated TauOs’ toxicity and mis-sorting, and the relationships between toxic AβOs and TauOs. The authors propose that soluble TauOs may be also responsible for seeding Tau pathology within AD brains, with a probable link to AβOs toxicity. After a critical summary of the effects of Tau on synaptic transmission and the toxicity of Tau aggregates, they compare the physiological functions of normal Tau and the toxic activity of pathological forms of Tau, highlighting the synaptic dysfunctions leading to impaired synaptic plasticity and activity, loss of the DNA-protective function of Tau, the loss of synapse and long-term potentiation in the hippocampus. Since the concentrations of TauOs in the cerebrospinal fluid (CSF) and plasma of AD patients were higher than in non-demented controls, the authors postulated that TauOs could be also considered as a CSF biomarker for the diagnosis of AD.

Intraneuronal AβOs accumulation precedes the appearance of amyloid plaques or neurofibrillary tangles and, at least in part, their toxic effects on neuronal survival, synaptic plasticity, and activity are due to the impairment of the function of calcium-binding proteins and calcium transport systems. Amyloid β1-42 (Aβ1-42) is the most abundant neurotoxic Aβ peptide in NFT and elicits cytosolic calcium homeostasis dysregulation at the early stages of brain degeneration in model animals of AD, as well as in neurons in culture. However, the AβOs-target proteins primarily lead to sustained alterations in intracellular calcium concentration, and neuronal calcium signaling pathways are still debated. The work of Poejo et al. [3] is focused on the hypothesis of the Aβ1-42 complexation, with calmodulin (CaM) as a molecular mechanism mediating calcium homeostasis dysregulation, and neuronal excitability alterations are widely accepted to play a key role in the early stages of AD. The micromolar concentration of CaM in neurons and its high affinity for neurotoxic Aβ peptides (dissociation constant ≈ 1 nM) highlight a novel function of CaM, i.e., the buffering of intracellular Aβ peptide concentrations in the low-nanomolar range. In turn, the concentration of Aβ-CaM complexes within neurons will increase as a function of time after the induction of Aβ peptides’ production. Thus, Aβ1-42:CaM complexation could also play a major role in neuronal calcium signaling mediated by calmodulin-binding proteins by Aβ, a point that has been overlooked to date. The implications of CaM complexation with neurotoxic AβOs in alterations in intracellular calcium homeostasis, as well as dysregulation of the calcium-dependent neuronal activity and excitability, are addressed in this review.

Tissue inflammation has been shown to play a major role in the development of tissue degeneration, not only in brain degenerative diseases but also in cardiovascular diseases. In neurodegenerative diseases, microglia activation secretes proinflammatory cytokines that induce the generation of neurotoxic reactive A1 astrocytes, and reactive astrocytes produce and release neurotoxic agents, such as Aβ peptides, among others. Reactive A1 astrocytes have been detected in post-mortem samples of AD, Huntington’s disease (HD), and other neurodegenerative diseases. However, the study of post-mortem samples cannot reveal whether these reactive A1 astrocytes are generated at early or later stages of the disease. In this Special Issue, Lopez-Sanchez et al. [4] show the early induction of reactive A1 astrocytes by the neurotoxin 3-nitropropionic acid (NPA) in rat brain, an animal model that mimics HD motor neurological dysfunctions. Of note, HD is also a recognized tauopathy. The results of this work demonstrated that intraperitoneal NPA injections elicit a significant increase in the expression levels of the C3α subunit, a marker of the generation of neurotoxic A1 astrocytes within the striatum, hippocampus, and cerebellum before the appearance of HD-related neurological dysfunctions and neuronal death induced by NPA. Notably, in the cerebellum, NPA administration primarily induces the generation of A1 astrocytes in the more recent phylogenetic area. It is concluded that the activation of complement C3 protein in the brain of Wistar rats is an early event in NPA-induced brain neurodegeneration.

Altered systemic factors are known to be relevant in many degenerative pathologies, including neurological and cardiovascular disorders. Cardiac sarcoidosis (CS) is a poorly understood disease and is characterized by the focal accumulation of immune cells, thus leading to the formation of granulomata (GL). GL consists of CD68+, CD163+ and CD206+ macrophages surrounded by T cells within fibrotic areas. Gajawada et al. [5] point out that the pathogenesis of sarcoidosis is still unclear, and its treatment options still cause controversial debates in the scientific and clinical fields. On these grounds, this article presents a fluorescence microscopy and Western blot analysis of CS and control patients, using antibodies to determine structural, cell cycle, and inflammatory markers. CS is macroscopically visualized by PET/computed tomography (CT). The phosphorylated ERM (ezrin, radixin, and moesin) and Erk1/2 proteins, strong expression of the myosin motor protein, and the macrophage transcription factor PU.1 are used as markers of highly active GL. Massive amounts of the IL-1R antagonist reflect a mild activation of stress and inflammatory pathways in GL. High levels of oncostatin M and the Reg3A and Reg3 chemokines are used as markers of macrophage accumulation in areas of remodeling cardiomyocytes. Their observations might help to explain why an anti-inflammatory corticosteroid therapy is beneficial at an early stage in patients with a preserved left ventricular function and is less effective in advanced cardiac sarcoidosis. The authors conclude that the formation of GL mainly occurs through chemoattraction and, to a lesser extent, by the proliferation of macrophages. They also propose that activation of the oncostatin/Reg3 axis might help to wall-off substances but might initiate chronic heart failure.

The molecular mechanisms underlying tissue homeostatic failure during aging will make them more prone to degeneration. In this Special Issue, Camello-Almaraz et al. [6] point out that aging not only modifies multiple cellular and homeostatic systems but also biological rhythms. Ambulatory recordings of the circadian rhythms of skin wrist temperature, motor activity, and the integrated variable temperature, activity, and position were dampened by aging, especially beyond 74 years of age. The authors focus on the circadian system driven by a central hypothalamic oscillator, which entrains peripheral oscillators. In both cases, these are underlined by circadian genes, and characterize the effect of aging in the circadian expression of clock genes in the human colon. A quantitative analysis of genes expressed in the muscle layer of colonic explants over 24 h revealed that the circadian expression of *Bmal1*, *Per1*, and *Clock* genes was larger beyond that age. For the first time, the authors have described the presence of a molecular oscillator in the human colon, which follows the pattern displayed by the amplitude of the molecular clock, i.e., an enhancement in elderly individuals.
